# Prótese Cardiaca Valvar Gravidez: Desafios e Estratégias

**DOI:** 10.36660/abc.20240602

**Published:** 2025-02-20

**Authors:** Marcelo Luis Nomura

**Affiliations:** 1 Hospital da Mulher José Aristodemo Pinotti Universidade Estadual de Campinas Campinas SP Brasil Área de Obstetrícia – Hospital da Mulher José Aristodemo Pinotti – Universidade Estadual de Campinas, Campinas, SP – Brasil

**Keywords:** Doenças Cardiovasculares, Gravidez, Mortalidade Materna

A gravidez em mulheres com próteses valvares é desafiadora para os cuidadores por vários motivos. Apesar dos avanços na compreensão da fisiologia cardiovascular, as intensas mudanças hemodinâmicas impostas pela gravidez em si e as interações entre a mãe, a placenta e o feto, juntamente com o ambiente único criado pela adaptação cardíaca, tornam o manejo uma das situações mais complexas para especialistas materno-fetais e cardiologistas. Adicione a isso a necessidade de anticoagulação eficaz e segura para a mãe e o feto para prevenir a maior ameaça de trombose e disfunção valvar.

Nesta edição, um amplo e elegante estudo observacional apresentou dados sobre os resultados perinatais de 128 gestações em mulheres com válvulas cardíacas protéticas.^1^ Como um estudo de centro único, os autores buscaram avaliar os resultados da gravidez e a taxa de complicações pós-parto de 12 meses e também comparar esses resultados entre válvulas cardíacas protéticas mecânicas e biológicas. Uma gravidez bem-sucedida foi definida por um parto a termo e puerpério sem intercorrências para a mãe e o recém-nascido e ocorreu em 50% dos casos. À primeira vista, pode-se olhar para isso como uma situação de copo meio cheio ou meio vazio. Em um olhar mais atento, no entanto, cerca de metade dos pacientes apresentaram complicações cardíacas, e um em cada três teve pelo menos um dos seguintes durante a gravidez: insuficiência cardíaca, fibrilação atrial, tromboembolismo ou endocardite infecciosa. Houve cinco mortes maternas, o que, por qualquer meio de comparação, é um número extremamente alto. Em relação aos resultados obstétricos, os principais achados foram uma alta taxa de aborto espontâneo no grupo de válvulas cardíacas protéticas mecânicas e também uma alta taxa de parto prematuro no grupo de válvulas bioprotéticas. A taxa geral de perdas fetais e neonatais foi de 30% (abortos espontâneos, natimortos e mortes neonatais).

Os números não falam por si. Os autores fizeram uma avaliação cuidadosa dos fatores de risco para resultados adversos. A disfunção da válvula cardíaca pré-concepcional, particularmente válvulas calcificadas bioprotéticas, foi fortemente associada à morbidade materna grave, incluindo morte. Pode parecer que as válvulas mecânicas tiveram um desempenho melhor. No entanto, os autores descrevem uma alta taxa de trombose valvar (uma levando à morte materna), que está associada a incertezas quanto ao regime de anticoagulação ideal para essas pacientes. O sangramento materno (pré-parto e pós-parto) é uma das complicações graves, embora não intencional.

A maior revisão publicada, com 499 gestações, mostrou menor taxa de complicações maternas e também baixa taxa de perdas perinatais.^2^ Mas talvez os resultados aparentemente melhores não possam ser estendidos à população brasileira, com alta incidência de gestações não planejadas e com características específicas, como maior incidência de etiologia reumática e falta de acesso a cuidados especializados.

À luz das descobertas do estudo, como podemos ajudar mulheres com válvulas cardíacas protéticas a terem melhores resultados para si mesmas e para seus filhos? É possível?

O aconselhamento pré-concepcional é uma das formas mais eficazes de melhorar os resultados da gravidez. A maternidade planejada pode potencialmente salvar vidas. Nesta população em particular, deve-se enfatizar que a gravidez não é aconselhada (na verdade, contraindicada) para mulheres com classificação classe IV da OMS ou com disfunção ventricular esquerda grave ou hipertensão pulmonar (classe funcional IV da NYHA) porque o risco de morte materna é muito alto.^3^ Para essas mulheres, a contracepção eficaz (e até mesmo a esterilização) deve ser discutida e fornecida de acordo com as preferências e segurança maternas. A contracepção não deve ser negligenciada. Também deve ser abordada com qualquer mulher com válvulas cardíacas protéticas, uma vez que gestações não planejadas representam riscos significativos para a mãe e o feto.^4^ Quem deve fazer isso? Cardiologistas e qualquer profissional de saúde com experiência na área não devem perder a oportunidade de levantar esta questão em qualquer consulta médica. Os ginecologistas devem estar preparados para fornecer opções seguras e eficazes.

Uma vez que uma mulher é aconselhada e decide engravidar, ela deve ser acompanhada de perto durante o processo de concepção, particularmente aquelas em uso de varfarina, sabidamente associada a abortos espontâneos e malformações fetais graves.

Cuidados especializados e multidisciplinares são de suma importância.^5^ Uma equipe experiente pode fornecer informações confiáveis e seguras e muitos recursos não disponíveis em outros níveis de cuidado. O acesso imediato a cuidados intensivos, cardiologia clínica e cirúrgica e assistência obstétrica e neonatal deve estar disponível.

Em condições ideais, que incluem cuidados pré-concepcionais cuidadosos e recursos humanos e materiais altamente qualificados disponíveis, a gravidez em mulheres com válvulas cardíacas protéticas não deve ser desencorajada (exceto nas situações citadas acima). No entanto, uma abordagem individualizada, fornecendo às mulheres informações confiáveis (como as que agora descrevemos neste estudo) sobre os riscos de morte materna e perda perinatal, é o mínimo, mas também o melhor que podemos fazer por elas.
