# Supplementary material for: Prótese Cardiaca Valvar Gravidez: Desafios e Estratégias
Source: Arq Bras Cardiol. 2025 Feb 20;122(1):e20240602. [Article in Portuguese] doi: 10.36660/abc.20240602 (PMC11834729; doi:10.36660/abc.20240602)
Supplement: Translation

# Translation: Cardiac Valve Prosthesis and Pregnancy: Challenges and Strategies

**DOI:** 10.36660/abc.20240602i

**Authors:** Marcelo Luis Nomura

**Affiliations:** 1 Brazil Área de Obstetrícia – Hospital da Mulher José Aristodemo Pinotti – Universidade Estadual de Campinas, Campinas, SP – Brazil

**Keywords:** Cardiovascular Diseases, Pregnancy, Maternal Mortality

Pregnancy in women with valvular prostheses is challenging for caregivers for several reasons. Despite advances in understanding cardiovascular physiology, the intense hemodynamic changes posed by pregnancy itself and the interactions between the mother, placenta, and fetus, along with the unique environment created by the cardiac adaptation, make management one of the most complex situations for maternal-fetal specialists and cardiologists. Add to it the need for effective and safe anticoagulation for both mother and fetus to prevent the greatest threat of valve thrombosis and dysfunction.

In this issue, a large observational and elegant study presented data on the perinatal outcomes of 128 pregnancies in women with cardiac prosthetic valves.^1^ As a single-center study, the authors sought to evaluate pregnancy outcomes and 12-month postpartum complications rate and also to compare these results between mechanical and biological prosthetic heart valves. A successful pregnancy was defined by an uneventful term birth and puerperium for both mother and newborn and occurred in 50% of the cases. At first glance, one might look at it as a half-full or half-empty glass situation. At a closer look, however, roughly half of the patients experienced cardiac complications, and one in three had at least one of the following during pregnancy: heart failure, atrial fibrillation, thromboembolism, or infective endocarditis. There were five maternal deaths, which, by any means of comparison, is an extremely high figure. Regarding obstetrical outcomes, the main findings were a high rate of spontaneous miscarriage in the mechanical prosthetic heart valves group and also a high rate of preterm birth in the bioprosthetic valve group. The overall fetal and neonatal loss rate was 30% (miscarriages, stillbirths, and neonatal deaths).

Numbers do not speak for themselves. The authors did a careful evaluation of risk factors for adverse outcomes. Preconceptional heart valve dysfunction, particularly bioprosthetic calcified valves, was strongly associated with severe maternal morbidity, including death. It might seem that mechanical valves had a better performance. However, the authors describe a high rate of valve thrombosis (one leading to maternal death), which is associated with uncertainties regarding the optimal anticoagulation regimen for these patients. Maternal bleeding (antepartum and postpartum) is one of the severe, although unintended, complications.

The largest review published, with 499 pregnancies, showed a lower rate of maternal complications and also a low rate of perinatal losses.^2^ But maybe the apparently better outcomes can not be extended to the Brazilian population, with a high incidence of unplanned pregnancies and with specific features, such as a higher incidence of rheumatic etiology and lack of access to specialized care.

In light of the study’s findings, how can we help women with prosthetic heart valves have better outcomes for themselves and their children? Is it possible?

Preconception counseling is one of the most effective ways to improve pregnancy outcomes. Planned motherhood could potentially save lives. In this particular population, it must be emphasized that pregnancy is not advised (in fact, contraindicated) for women with class IV WHO classification or with severe left ventricular dysfunction or pulmonary hypertension (NYHA functional class IV) because the risk of maternal death is too high.^3^ For these women, effective contraception (and even sterilization) must be discussed and provided according to maternal preferences and safety. Contraception must not be neglected. It should also be addressed with any women with prosthetic heart valves since unplanned pregnancies pose significant risks for the mother and fetus.^4^ Who should do this? Cardiologists and any care provider with expertise in the field must not lose the opportunity to bring up this issue at any office visit. Gynecologists must be prepared to provide safe and effective options.

Once a woman is counseled and decides to get pregnant, she must be closely followed during the process of conception, particularly those in use of warfarin, knowingly associated with miscarriage and severe fetal malformations.

Specialized and multidisciplinary care is of the utmost importance.^5^ An experienced team can provide reliable and safe information and many resources not available at other levels of care. Immediate access to intensive care, clinical and surgical cardiology, and obstetric and neonatal assistance must be available.

Within ideal conditions, which include careful preconception care and highly skilled human and material resources available, pregnancy in women with prosthetic heart valves should not be discouraged (except for the situations cited above). However, an individualized approach, providing women with reliable information (such as we have now depicted in this study) about risks of maternal death and perinatal loss, is the minimum but also the best we can do for them.
